# Dynamic pH and Thermal Analysis of Paper-Based Microchip Electrophoresis

**DOI:** 10.3390/mi12111433

**Published:** 2021-11-22

**Authors:** Muhammad Noman Hasan, Ran An, Asya Akkus, Derya Akkaynak, Adrienne R. Minerick, Chirag R. Kharangate, Umut A. Gurkan

**Affiliations:** 1 Department of Mechanical and Aerospace Engineering, Case Western Reserve University, Cleveland, OH 44106, USA; muhammad.hasan@case.edu (M.N.H.); asya.akkus@case.edu (A.A.); crk91@case.edu (C.R.K.); 2 Department of Ecology & Evolutionary Biology, Princeton University, Princeton, NJ 08544, USA; derya.akkaynak@gmail.com; 3 Department of Chemical Engineering, Michigan Technological University, Houghton, MI 49931, USA; minerick@mtu.edu; 4 Department of Biomedical Engineering, Case Western Reserve University, Cleveland, OH 44106, USA

**Keywords:** paper-based electrophoresis, pH shifts, temperature shifts, hemoglobin separation

## Abstract

Paper-based microchip electrophoresis has the potential to bring laboratory electrophoresis tests to the point of need. However, high electric potential and current values induce pH and temperature shifts, which may affect biomolecule electrophoretic mobility thus decrease test reproducibility and accuracy of paper-based microfluidic electrophoresis. We have previously developed a microchip electrophoresis system, HemeChip, which has the capability of providing low-cost, rapid, reproducible, and accurate point-of-care (POC) electrophoresis tests for hemoglobin analysis. Here, we report the methodologies we implemented for characterizing HemeChip system pH and temperature during the development process, including utilizing commercially available universal pH indicator and digital camera pH shift characterization, and infrared camera characterizing temperature shift characterization. The characterization results demonstrated that pH shifts up to 1.1 units, a pH gradient up to 0.11 units/mm, temperature shifts up to 40 °C, and a temperature gradient up to 0.5 °C/mm existed in the system. Finally, we report an acid pre-treatment of the separation media, a cellulose acetate paper, mitigated both pH and temperature shifts and provided a stable environment for reproducible HemeChip hemoglobin electrophoresis separation.

## 1. Introduction

Paper-based microfluidics provide low-cost, easy-to-use and portable bio-sensing solutions for a broad spectrum of point-of-care (POC) applications, including disease screening/diagnosis, environmental monitoring, food safety, water testing, as well as drug screening and delivery [1,2,3]. Implementation of these paper-based POC platforms can reduce the needs of sophisticated laboratory infrastructure, expensive reagents, and skilled personnel, which are often lacking in low-resource settings [1]. Paper-based electrophoresis microfluidic platforms were introduced in 2014 and have been utilized to separate and detect various target molecules, including serum proteins, certain amino acids, bovine hemoglobin, and cytochrome C [4,5,6].

Electrophoretic separation of sample components is an essential step in analytical chemistry, biochemistry, as well as for disease diagnosis. The electric field that drives electrophoresis also drives electrolytic reactions at both electrode surfaces that manifest as water electrolysis (Equations (1) and (2)) which have side effects, including generation of gas bubbles and induction of pH shifts at both the anode and cathode [7].

Anode: 2H_2_O ⇌ O_2_(g) + 4H^+^ + 4e^−^ (1)

Cathode: 2H_2_O + 2e^−^ ⇌ H_2_(g) + 2OH^−^ (2)

In addition to the pH shift, temperature changes induced by joule heating from the electric current passing through the separation buffer also exist during electrophoresis (Equation (3)), where σ is the conductivity of electrophoresis separation medium (S/m) and E is the electric field (V/m) [8].

(3) $$\Delta T=\sigma E^{2}$$

In traditional electrophoresis systems or glass-based microfluidic electrophoresis, these electrochemical reactions, pH changes, and joule heating effects have been well characterized and can be partially controlled to achieve a more stable separation environment [9,10,11,12,13,14,15,16]. Multiple strategies have been developed to mitigate these effects, including employing large-volume liquid reservoirs around the electrodes, involving continuous fluid flow of the buffer solution, as well as adding chemical components to the buffer. However, these strategies are often difficult to employ in miniaturized devices since most of them require large reservoirs or complicated flow systems. Additionally, these pH and temperature shifts have not been fully characterized in paper-based electrophoresis microfluidic systems such as our HemeChip, thus, some of these technologies are at risk of experiencing compromised separation performance, reproducibility, and robustness under higher electric potentials.

We have previously developed the first mass-producible, paper-based microchip electrophoresis (HemeChip) system for separation, identification, and quantification of various subtypes of hemoglobin variants for screening and diagnosing hemoglobin disorders (e.g., sickle cell disease) [7,17,18,19,20,21,22,23]. Particularly in sickle cell disease (SCD), polymerization of hemoglobin S makes red blood cells stiff, which triggers abnormal cellular adhesion on inflamed vascular walls and the hallmark vaso-occlusive crisis [24,25,26,27,28]. HemeChip enables newborn/neonatal screening and, thus, early, comprehensive medical care to mitigate SCD-related complications. HemeChip utilizes a strip of cellulose acetate (CA) paper as the separation medium and high electric potentials of around 200 V are applied. In this work, we report the methodologies we implemented for characterizing HemeChip system pH and temperature during the development process, including utilizing commercially available universal pH indicator and digital camera pH shift characterization, and infrared camera characterizing temperature shift. The characterization results demonstrated that pH shifts up to 1.1 units, a pH gradient up to 0.11 units/mm, temperature shifts up to 40 °C, and a temperature gradient up to 0.5 °C/mm existed in the system. Additionally, we report one approach that we employed to mitigate the pH and temperature shifts in our paper-based microchip electrophoresis system.

## 2. Materials and Methods

### 2.1. Material

CA paper was purchased from VWR (Radnor, PA, USA). The electrophoresis buffer, Tris/Borate/EDTA (TBE) 10×, and the ultrapure water were purchased from ThermoFisher Scientific (Waltham, MA, USA). Universal pH indicator (UPI) was purchased from Sigma-Aldrich (St. Louis, MO, USA). Standard pH solution (colorless) with pH values of 5.0, 5.5, 6.0, 6.86, 7.0, 8.0, and 10.0 were purchased from Fisher Scientific (Hampton, NH, USA) and the standard pH solution with pH values of 8.0 and 9.0 were purchased from Thomas Scientific (Swedesboro, NJ, USA). Raspberry Pi (RPi) 3B+ camera was purchased from MicroCenter (Cleveland, OH, USA). Micro color checkerboard (ColorGauge Micro, Matte) was purchased from Edmund Optics (Barrington, NJ, USA). Handheld smartphone interfaced infrared camera (FLiR One) and infrared image analysis software were purchased from FLiR Systems (Wilsonville, OR, USA). K-type thermocouple (bead-probe, −200 °C~750 °C) and the 1/4 DIN Temperature/Process Controllers (used as a thermocouple reader, resolution 0.1 °C) were purchased from Omega Engineering (Norwalk, CT, USA). Silicone-based thermal interface material (TIM) was purchased from Grainger Industrial Supply (Brooklyn Heights, OH, USA).

### 2.2. HemeChip Cartridge and Reader

**Figure 1:**
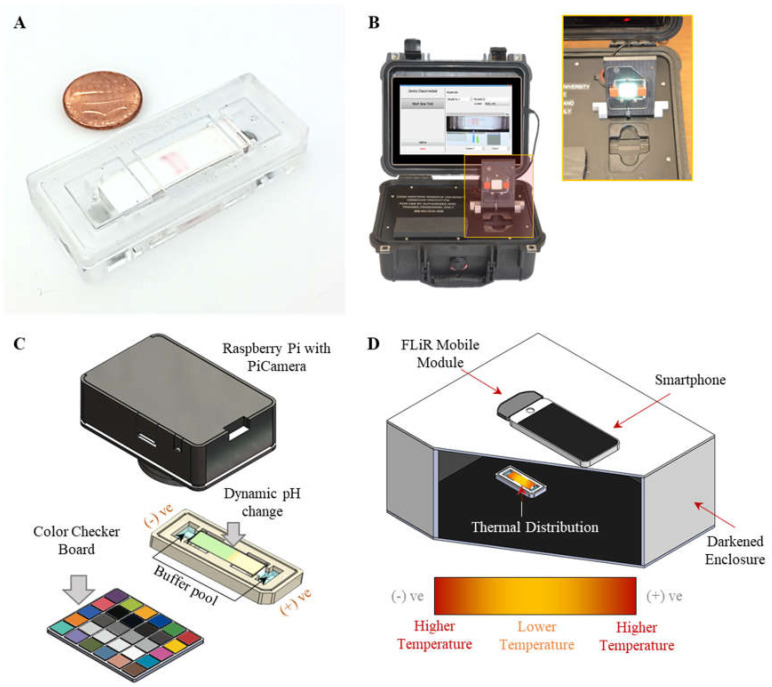
System setup for dynamic run-time pH and temperature-change tracking in the HemeChip cartridge. (**A**) An injection molded mass-produced HemeChip cartridge. (**B**) The HemeChip reader contains the hardware, software, and a user interface to guide the user through the test and display test result at the end of the test. (**C**) Illustration of the dynamic pH monitoring system on CA paper mounted on the HemeChip cartridge. (**D**) Illustration of the dynamic temperature-monitoring system on CA paper.

The HemeChip cartridge is a single-use electrophoresis microchip (Figure 1A) [18]. Briefly, the cartridge is composed of injection molded plastic parts made of Optix^®^ CA-41 Polymethyl Methacrylate Acrylic (Thogus Products, OH, USA) which contain two buffer reservoirs filled with 200 µL buffer each. The HemeChip cartridge embodies a pair of round corrosion-resistant, biomedical grade stainless-steel 316 electrodes (Med-Tex, USA); and a piece of cellulose acetate paper with plastic-sheet support (Radnor, PA, USA). The HemeChip reader is a battery powered, portable platform for running the HemeChip test in remote locations and/or in resource-limited settings (Figure 1B). The HemeChip reader and the cartridge were customized in this study for the dynamic measurement of pH and temperature change (Figure 1C,D). 

### 2.3. Dynamic Track of pH Shift in Microchip Electrophoresis

The electrophoretic running buffer (Tris-borate-EDTA, TBE) was prepared from the 10× UltraPure^TM^ TBE buffer stock solution (ThermoFisher). For tracking of dynamic pH distribution in the paper-based microchip electrophoresis system, 1× modified TBE buffer was prepared by diluting 10× stock solution in the pre-prepared water solution of UPI. pH calibration buffers were prepared by diluting UPI into an array of pH standard solutions, from pH 5.0 to pH 10.0. 

**Figure 2:**
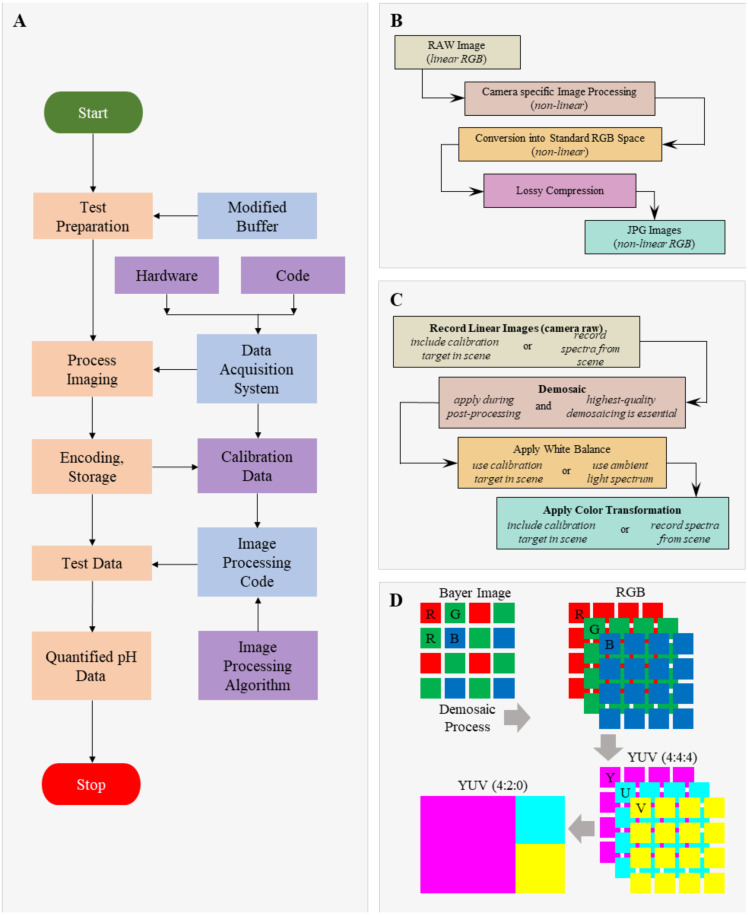
Flow-chart of data analysis for pH measurement. (**A**) Process flow-chart. (**B**) Image-processing algorithm for lossy compression. (**C**) Image-processing algorithm for loss-less processing. (**D**) Conversion of sensor data into YUV420 planar data format. This uncompressed linear format prevents any loss of data required for the image analysis.

**Figure 3:**
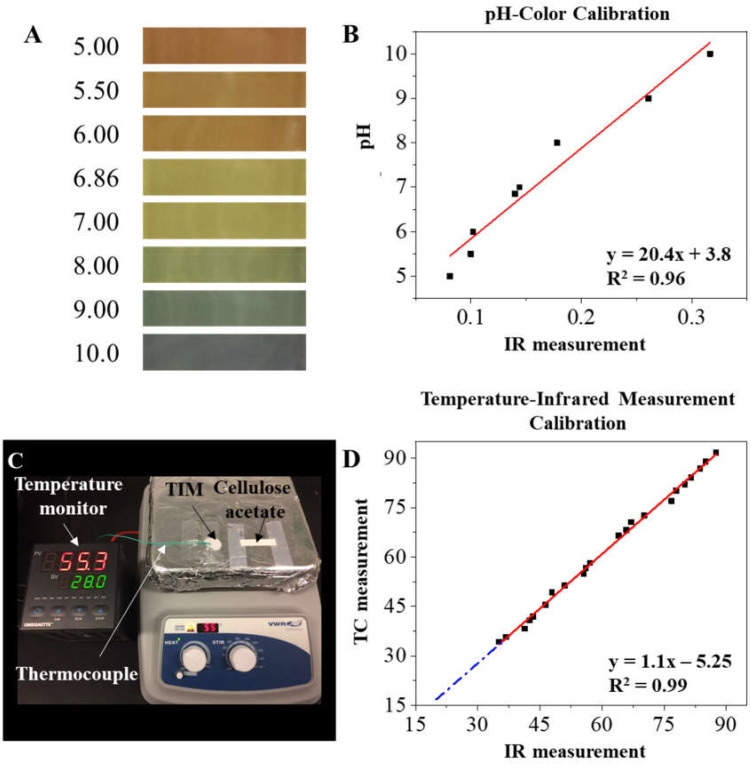
Calibration of pH and temperature-monitoring systems. (**A**) Images of the calibration system for monitoring pH change: CA paper with buffer containing pH indicator at given pH values (pH = 5 to pH = 10) were imaged. These imaged data were used to create the pH calibration curve demonstrated in (**B**). (**C**) Image of calibration system for temperature-change monitoring: CA paper is heated on the hot plate and the infrared temperature measurement is performed using images captured by the FLiR Studio software. The actual temperature is measured from the reading of a thermocouple (K-type) mounted to the hot plate. Infrared temperature measurements are calibrated to the thermocouple temperature measurements (**D**).

During each test, the CA paper strip was first wetted with the modified TBE buffer and placed into the micro-cartridge. The same modified TBE buffer was then filled into both buffer reservoirs at each end of the cartridge. To convert calorimetric changes into pH values, we implemented an affordable digital camera (Eplcctv, Shenzhen, China) to perform image acquisition with a standard color checkerboard in real time at 1 frame per second (Figure 1C). Dynamic pH changes were evaluated at multiple applied voltages of 50, 150, and 250 V (corresponding electric-field strength at 1667, 5000, and 8333 V/mm). The acquired uncompressed raw and linear image data (YUV420) was used to ensure measurement accuracy (Figure 2). The process of image acquisition, calibration, and the interpretation is outlined in Figure 2A. Careful considerations were made to avoid lossy compression and to ensure the linearity (Figure 2B) of the captured and stored image format (Figure 2C). The pH calibration curve was developed to convert the calorimetric information (on the separation medium) to pH values using prepared pH standard solutions (Figure 3A,B).

### 2.4. Dynamic Tracking of Temperature Shifts in Microchip Electrophoresis

The infrared imaging system consisted of a dual-camera (FLiR One) which was connected with a smartphone for performing temperature measurement in a customized darkened environment to prevent an effect from ambient light (Figure 1D). The infrared imaging system was first calibrated to eliminate the effects of CA paper-surface emission and the air relative humidity (Figure 3C,D). Briefly, wetted CA paper was attached on top of a plate heater at temperatures from 35 to 90 °C. A K-type thermocouple was attached on the plate heater with TIM placed between the heated surface and the thermocouple probe to precisely monitor the actual temperature of the CA paper. The CA paper was then imaged using the FLiROne camera from 35 to 90 °C to generate the calibration curve (Figure 3D). 

### 2.5. CA Paper Pre-Treatment

We also characterized pH and temperature change over the CA paper after pre-treatment using a customized method. The purchased CA papers were submerged into a diluted hydrochloric acid, HCl (pH 4.0) for 30 min. After 30 min, the CA papers were removed from the solution and allowed to air dry at room temperature overnight. The dry CA papers were then laser-cut into the required dimensions and used for running HemeChip tests.

## 3. Result and Discussion

### 3.1. pH and Temperature-Tracking System Calibration

#### 3.1.1. pH-Tracking System Calibration

CA paper wetted with prepared pH standard solution was used to generate the calibration curve used to convert the color in captured images to corresponding pH values (Figure 3B). The calibration curve provides a linear relationship between the color parameter, hue (x-axis) and the pH value (y-axis) with a fit:

pH value = 20.4 × Hue value + 3.8, (R^2^ = 0.96) (4)

#### 3.1.2. Temperature-Tracking System Calibration

The infrared image data acquired during the experiment were calibrated with the thermocouple reading for thermal analysis. The temperature directly read by the infrared imaging system demonstrated comparable but slightly deviated data from the standard thermocouple-based temperature monitor. This deviation could be caused by relative air humidity, or the internal IR detection variance of the FLiROne device as well as surface emissivity of the CA paper while imaged. As a result, a calibration curve (Figure 3D) was obtained to provide accurate association between the temperature directly read by the infrared imaging system to the actual temperature measured by the thermocouple, as described in Equation (5):

Temperature (Actual) = 1.1 × Infrared Measured Temperature−5.25, (R^2^ = 0.99) (5)

### 3.2. pH Shift during Paper-Based MicroChip Electrophoresis

**Figure 4:**
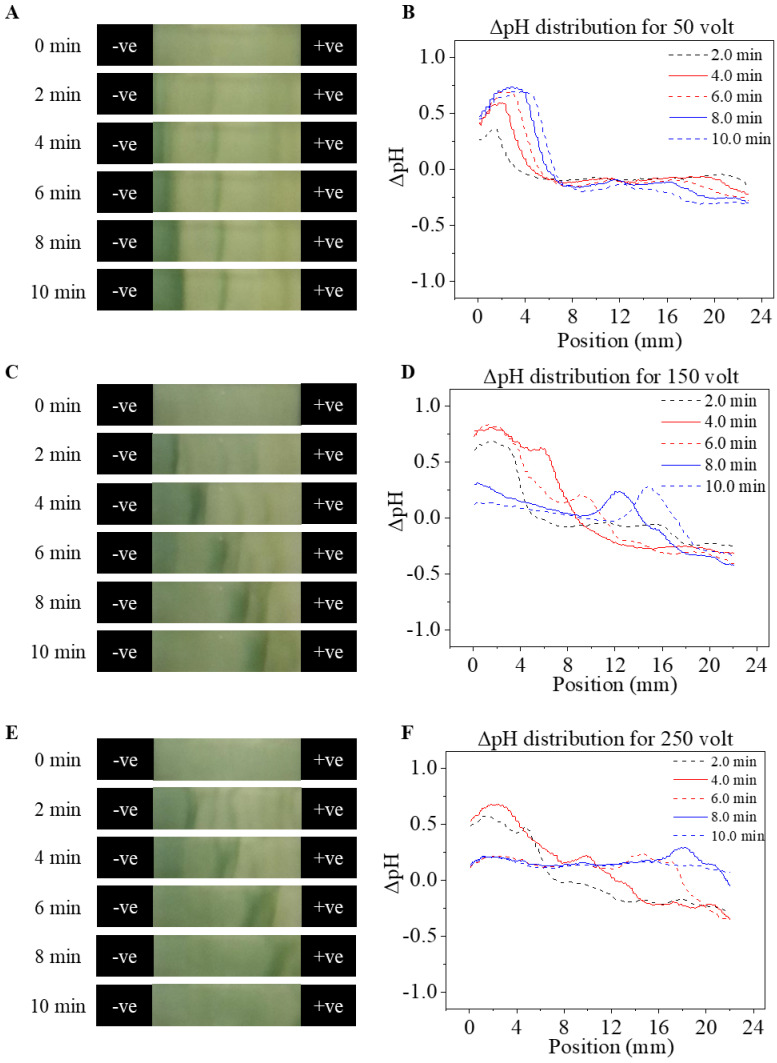
Spatial pH distribution at 0, 2, 4, 6, 8, and 10 min under different electric fields. (**A**,**B**) Time-lapse images and pH distribution on CA paper under 1667 V/m (50 V). (**C**,**D**) Time-lapse images and pH distribution on CA paper under 5000 V/m (150 V). (**E**,**F**) Time-lapse images and pH distribution on CA paper under 8333 V/m (250 V).

Figure 4 demonstrates the pH change across the CA paper within 10 min runtime under 16.67 V/cm (Figure 4A,B), 5000 V/m (Figure 4C,D), and 8333 V/m (Figure 4E,F) applied voltages. The left column in Figure 4 includes unprocessed captured images from the central region of the CA paper (from 4 to 26 mm of the 30 mm long CA paper as well as the entire y-axis width (9.5 mm) of the CA paper). The right column in Figure 4 includes the pH profile averaged over the y-axis of the CA paper plotted against the x-axis position (mm). 

Uniform pH distributions were detected in all three sets of tests at the 0 min time before application of the electric potential. The semi-quantitative measurement demonstrated that at all applied potentials of 1667 V/m, 5000 V/m, and 8333 V/m, the pH started to increase on the cathode side and decrease at the anode side within a short time (2 min) of exposure to the electric field. Under lower field strength of 1667 V/m (Figure 4A,B), the absolute magnitude of the pH shifts at both ends of the CA paper were lower (~1-unit increase on the anode side and about 0.5 unit decrease on the cathode side), which resulted in a pH gradient up to 0.05 unit/mm. In addition, the absolute magnitude of the pH shifts gradually increased monotonically over time 0 to time 10 min near both electrodes. However, the detected pH value in the central region of the paper (4–16 mm) only shifted slightly (<0.25 unit). Under stronger electric fields of 5000 V/m (Figure 4C,D) and 8333 V/m (Figure 4E,F), higher absolute magnitudes of pH shifts were observed near both electrodes. Additionally, the areas in which pH shifted also expanded from both electrodes to the central bulk region of the CA paper, causing an increment of 0.5 units in pH value which generated an average pH gradient of 0.11/cm.

The pH shifts were induced by electrochemical reactions (Equations (1) and (2)) initiated on surfaces of electrodes at both ends of the CA paper [18]. Excessive hydrogen ions were generated in solution closer to the anode surface while excessive hydroxide ions were generated in solution closer to the cathode surface. As time progressed, the excess hydrogen and hydroxide ions then migrated toward the bulk portion of the media (CA paper) under the combined effects of diffusion and electromigration [29], resulting in the expanded area of pH shift. Biomolecule electrophoretic mobilities are determined by the charge-to-mass ratio and the net charge on the molecule, which is strongly affected by the pH of its surrounding environment [9]. For example, normal hemoglobin (HbA) demonstrates a net charge of −14 at pH 8.6, while at pH 12, the HbA demonstrates a net charge of −67 [30]. Uniform and stable pH is the foundation for reproducible biomolecular electrophoresis [9,10,11,12,13,14,15,16]. As a result, the pH shifts and pH gradients observed here, if not mitigated, can potentially alter the process and characteristics of the hemoglobin electrophoretic separation. This may raise the risk of compromising consistent and accurate identification of each hemoglobin subtype, which further affects the accuracy and reproducibility of the HemeChip system.

### 3.3. Temperature Shift during Paper-Based MicroChip Electrophoresis

**Figure 5:**
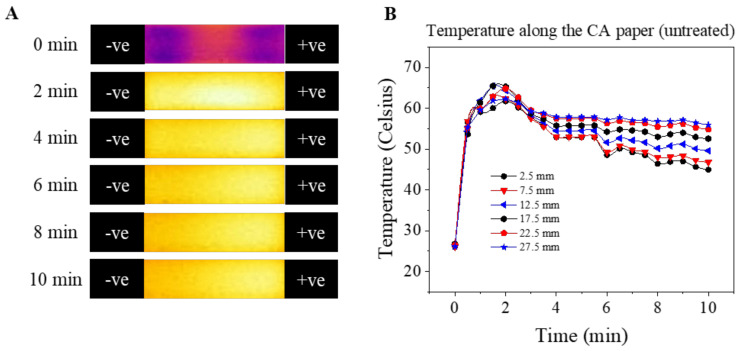
Spatial temperature distribution at 0, 2, 4, 6, 8, and 10 min under electric field. (**A**) Time-lapse infrared images of a CA paper run under 8333 V/m (250 V) electric field. (**B**) Average temperature change with time of the CA paper at 2.5 mm, 7.5 mm, 12.5 mm, 17.5 mm, 22.5 mm, and 27.5 mm from the anode under 8333 V/m (250 V) electric field. Temperature changes up to 40 °C (absolute temperature >65 °C) were reached within 2 min after applying the electric field. The magnitude of the temperature changes (ΔT) then reduced to 20 to 30 °C depending on the x-axis position. Overall, higher ΔT were observed at positions closer to the anode than at positions closer to the cathode side of the CA paper.

Figure 5 demonstrates the temperature shift across the CA paper at 0, 2, 4, 6, 8, and 10 min under 8333 V/m. Figure 5A includes the unprocessed captured images from 4 to 26 mm of a 30 mm long CA paper as well as the entire y-axis width of the CA paper (9.5 mm). The electrode gap is 30 cm, but the HemeChip hemoglobin separation occurs from 4 to 26 mm. Figure 5B includes the measured y-axis-averaged temperature change over time at various x-axis positions on the CA paper (from 2.5 mm to 27.5 mm, with 5 mm intervals). 

Temperature shifts and spatial temperature gradients were observed over the x-axis of the CA paper at almost all time points after exposure to the electric field, with the anode side having a higher temperature relative to the cathode side. The amplitude of absolute temperature shifts, ΔT, increased from 0 to 120 s from 0 up to 40 °C (absolute temperature, T, from 25 to 65 °C) on both ends of the electrodes and decreased to ~38.5 °C (absolute temperature, T = 63.5 °C) from 120 s to 320 s on the anode side and ~25 °C (T = 50 °C) from 120 s to 360 s on the cathode side (Figure 5B). The magnitude of the spatial temperature gradient between the anode to the cathode increased with time (0 °C/mm at t = 0, 0.2 °C/mm at t = 120 s, and 0.5 °C/mm at t = 600 s, Figure 5B). 

In the paper-based microchip electrophoresis system, the temperature change was associated with: (1) joule heating generated from the electric current passing through the buffer soaked CA paper [15,16]; (2) electrochemical reactions related to heat consumption (endothermic water electrolysis reaction) [16]; (3) heat dissipation induced by water phase change (vaporization) [31,32]; and (4) passive heat dissipation by conduction/diffusion [31,32]. The initial temperature raises between 0–120 s were mainly attributed to joule heating induced by the conductive buffer solution and applied electric field. The decrease in temperature temporally from 120–600 s (Figure 5B) was probably due to: (a) the elevated magnitude of heat diffusion induced by the high temperature gradient (~41 °C from CA paper to room temperature of 25 °C); and (b) the reduced rate of heat generation by joule heating due to the reduction in electric current. This reduction in electric current is probably due to the stabilization of the electrochemical system, as described previously in other systems [33]. With time, the system reached a quasi-equilibrium state where the total generated heat was in balance with the heat dissipated by the system. 

Biomolecule electrophoretic mobilities are determined via ion dissociation constant and solution viscosity, both of which are functions of temperature [34,35]. As a result, uncontrolled temperature shifts can cause non-ideal behavior during electrophoresis, including experimental variability, joule-heating-induced zone dispersion, and increased electromigration dispersion [34]. The temperature shifts and temperature gradients observed in this work, if not mitigated, can also alter the progression and characteristics of hemoglobin electrophoretic separation. This has the potential to compromise the capability of consistent and accurate identification of each hemoglobin subtype, further affecting the accuracy and reproducibility of the HemeChip system.

### 3.4. Mitigation of pH and Temperature Shifts

**Figure 6:**
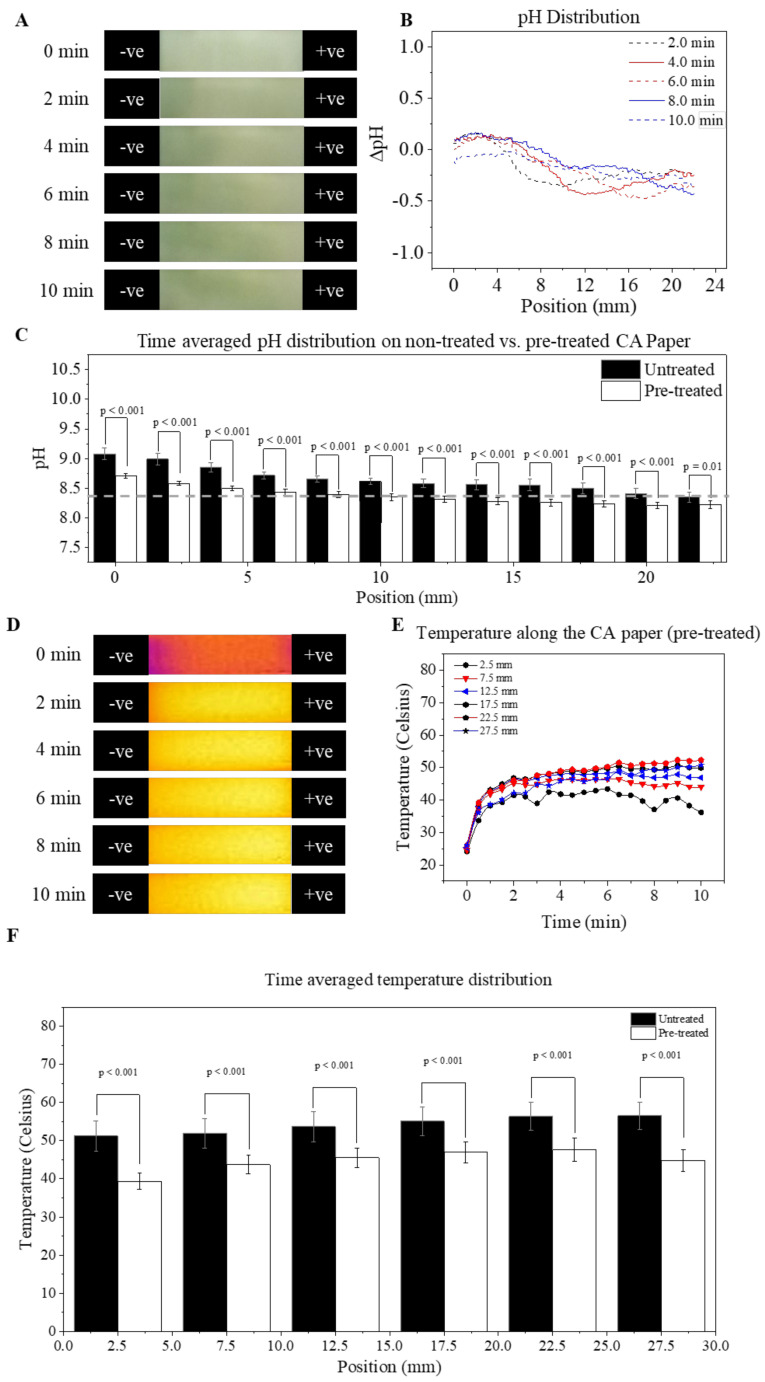
Thermal mapping of the pre-treated CA paper. (**A**) time-lapse of a HemeChip test run at 83.33 V/cm with a pre-treated CA paper, (**B**) pH distribution of a HemeChip test run at 8333 V/m with a pre-treated CA paper, (**C**) time-averaged pH distribution comparison between a treated and non-treated CA paper, (**D**) temperature distribution of a HemeChip test run at 83.33 V/cm with a pre-treated CA paper, (**E**) yaxis-averaged temperature distribution along the x-axis of a pre-treated CA paper, and (**F**) time averaged temperature distribution comparison between an untreated and a pre-treated CA paper.

Stable environments with constant pH and temperature are fundamental for reproducible electrophoretic separations. Due to the observed non-ideal pH and temperature shifts above, we sought methods to mitigate these effects and discovered that pre-treatment of CA paper using HCl was an effective approach for our application. Figure 6 demonstrates the effect of this pre-treatment approach on mitigating pH and temperature shifts during the paper-based microchip electrophoresis. Relatively stable pH values were observed. Lower absolute pH shifts within the range of −0.5 to 0.3 were observed on pre-treated CA paper over a 600 s time period within a 8333 V/m electric field (Figure 6A,B). Compared to the non-treated CA paper (black filled bar), the pre-treated CA paper (open bar) demonstrate significant reduction (*p* < 0.001) in time averaged pH shifts from the desired pH 8.4 (dashed gray line) on the entire length of the CA paper (Figure 6C).

Pre-treatment of the CA paper also facilitated a mitigation of the temperature shift during electrophoresis. Under a 8333 V/m electric field, the temperature on the pre-treated CA paper monotonically increased with time, with the maximum temperature shift of 27.5 °C (T = 52.5 °C) observed at the end of the test (600 s, Figure 6D,E). The time-averaged temperature at each spatial location on the pre-treated CA paper was also significantly reduced from the non-treated paper (treated: 39.7 °C–46.3 °C vs. untreated: 51.0 °C–58.9 °C, *p* < 0.001). Overall, the implemented pre-treatment approach mitigated shifts in both pH and temperature within the system throughout the entire electrophoresis runtime. The reduced pH and temperature shifts provided an environment with enhanced stability for hemoglobin electrophoresis, and improved test reproducibility and accuracy. The physical or chemical effect of this CA paper pre-treatment method has not been the focus of this work and remains unclear. One possibility is that the HCl dynamically reduced the free ions in the buffer. The paper acted as an ion sink, thus stabilizing the pH. The secondary impact of reducing free ions is a reduction in current and, thus, joule heating. Another possibility is that the acid treatment could have reduced the resistance of the CA paper. The reduced CA paper resistance would then reduce the total resistance of the CA paper-separation media combination, therefore the voltage drop (electric field) across the separation media within the paper decreased while the voltage drop (electric field) across the buffer in both reservoirs increased. This reduction in electric field then led to reduced joule heating, according to Equation (3). However, this mechanistic understanding of the physical or chemical effect of acid treatment on CA paper has not been the focus of this work, therefore they require further investigation.

## 4. Conclusions

Stable and well-controlled pH and temperature are fundamental for generating accurate and reproducible data from paper-based electrophoretic systems. This study reports a simple and affordable method we implemented to characterize pH and temperature shifts on the CA paper for our paper-based microchip electrophoresis system. The results demonstrated temporal shifts in pH and temperature, as well as formation of spatial pH gradient and temperature gradient over native CA paper induced by electrochemical reaction and joule heating, respectively. These shifts in buffer properties can alter protein electromigration behaviors, thus increasing the risk of compromised protein detection reproducibility and accuracy. Pre-treatment of the CA paper using diluted HCl mitigated temporal shifts for both pH and temperature, as well as decreased the spatial pH gradient. The pre-treated CA paper demonstrated improved pH and temperature stability compared to the non-treated paper through the test runtime of 600 s, even under a relatively high electric field of 8333 V/m. In our previous publication, the HemeChip system that implemented these pre-treated CA paper demonstrated high stability and accuracy in detecting hemoglobin variant via testing 768 subjects at multiple clinical sites [18]. These high-accuracy field-test results reflect that the pre-treatment of CA paper provided a stable and beneficial environment for hemoglobin electrophoresis in our paper-based microchip electrophoresis system. The mechanism of how this particular pre-treatment approach mitigates the spatial and temperature requires further investigation to strengthen the current understanding. Further work will be pursued to understand the physics behind this effect, probably on the physical and/or chemical properties of the CA paper.

## Data Availability

All reasonable requests for materials and data will be fulfilled by the corresponding author of this publication.
